# Synthetic explorations of the briarane jungle: progress in developing a synthetic route to a common family of diterpenoid natural products

**DOI:** 10.1098/rsos.172280

**Published:** 2018-05-23

**Authors:** Nicholas G. Moon, Andrew M. Harned

**Affiliations:** 1 Department of Chemistry, University of Minnesota—Twin Cities, 207 Pleasant Street SE, Minneapolis, MN 55455, USA; 2 Department of Chemistry and Biochemistry, Texas Tech University, 1204 Boston Avenue, Lubbock, TX 79409-1061, USA

**Keywords:** natural products, diterpenoids, total synthesis, stereoselectivity, conformational analysis

## Abstract

The briarane diterpenoids are a large family of marine natural products that have been primarily isolated from gorgonian octocorals around the world. Structurally, the family is characterized by a *trans*-fused bicyclo[8.4.0]tetradecane ring system containing a central, stereogenic, all-carbon quaternary carbon (C1) flanked by three additional stereocentres (C2, C10, C14). Many family members have demonstrated biological activity in numerous areas, including: cytotoxicity, anti-inflammatory, antiviral, antifungal, immunomodulatory and insect control. Despite their interesting structural properties and bioactivity, the briaranes have been largely overlooked by the synthetic community. However, in recent years, several research groups have reported progress toward developing a synthetic route to these natural products. Most of these efforts have focused on the stereoselective construction of the central C1–C2–C10–C14 stereotetrad. This review will discuss the various synthetic efforts aimed at the briarane diterpenoids along with the challenges that remain.

## 1. Introduction and overview of the briarane diterpenoids

**Figure 1.**
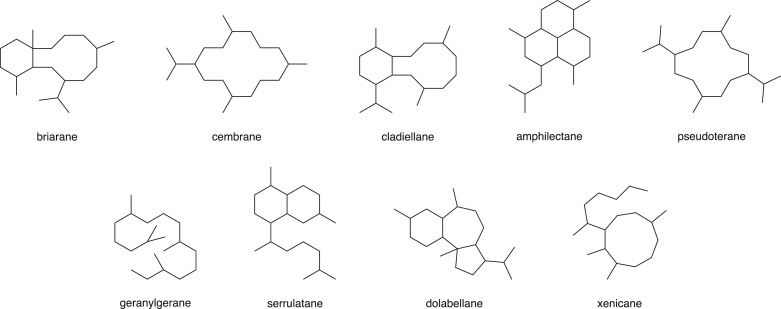
The nine most common diterpenoid skeletons isolated from the gorgonian family.

Marine gorgonians represent a diverse set of octocoral organisms that dominate coral reefs throughout the world. The gorgonians can be further divided into three orders: Scleraxonia, Holaxonia and Calaxonia. Owing to the corals' natural abundance and wide geographical distribution, the gorgonians have been a fruitful area of study for natural products chemists [1], and the chemical makeup of a particular gorgonian sample has often served as a taxonomic marker for gorgonian classification [2]. In this regard, diterpenoids represent one of the more important classes of natural products that have been isolated from gorgonian sources. In 2009, Berrue & Kerr discussed the isolation of 40 different diterpenoid skeletal structures from gorgonians [1]. Figure 1 shows the nine most common skeletal structures.

**Figure 2.**
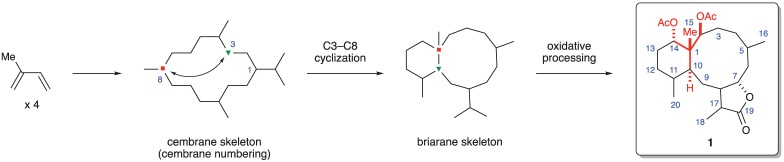
Proposed biosynthesis of the briarane skeleton.

Briarane-type diterpenoids represent one of the more common natural product families to be isolated from gorgonian octocorals. The isolation of the first family member (briarein A) was reported in 1977 [3]. Since that time, over 700 unique briarane diterpenoids have been characterized [4–9]. Briarane-type diterpenoids have been isolated from both the Scleraxonia and Calaxonia suborders, but not the Holaxonia suborder. The family Briareidae (suborder Scleraxonia) is by far the most plentiful source of briarane diterpenoid compounds. In addition to gorgonian sources, several examples have also been reported from non-gorgonian sources such as the sea pansy *Renilla reniformis* [10], the sea pen octocoral *Scytalium tentaculatum* [11] and the Mediterranean nudibranch mollusk *Armina maculata* [12]. It has been hypothesized [4–9] that the briaranes are biosynthetically derived from the cembrane skeleton, another commonly found skeletal structure in marine gorgonian species, through cyclization between C3 and C8 followed by further oxidative processing (figure 2). However, no biosynthetic studies have been published.

**Figure 3.**
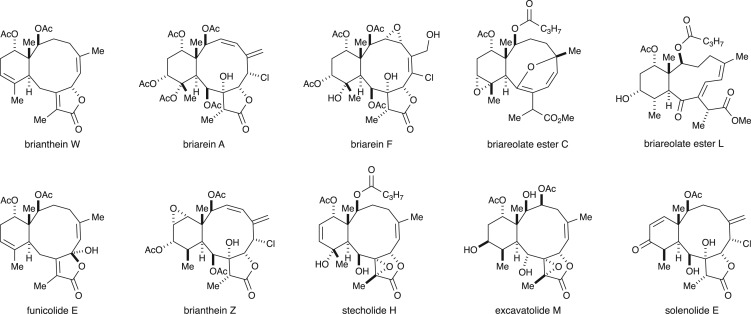
Representative members of the briarane family.

In their final form (**1**), briarane diterpenoids are characterized by a *trans*-fused bicyclo[8.4.0]tetradecane ring system. Most members also possess a γ-lactone spanning C7 and C8. Another common feature is a congested set of four, contiguous stereogenic centres comprising C1–C2–C10–C14. Oxidative processing by the organism can install oxygenation at nearly every carbon in the system, as evidenced by the representative family members shown in figure 3 [4–9].

Given the size and structural diversity associated with the briarane family, it is not surprising that many family members have demonstrated biological activity in a number of areas including: cytotoxicity, anti-inflammatory, antiviral, antifungal, immunomodulatory, insect control, antifouling and ichthyotoxicity [4–9,13]. Despite the wide structural diversity and interesting biological activity associated with the briaranes, relatively little effort has been expended in devising synthetic routes to access these natural products. Only a few groups have reported synthetic progress towards any member of the briaranes. To date, no completed total synthesis has been reported; a fact that illustrates the significant challenge posed by these targets. These efforts will be the subject of this mini-review. Briarane numbering of carbon atoms will be used throughout this review.

## 2. Early synthetic approaches

This section will discuss early reports that were focused on constructing various small or highly simplified fragments, including the 10-membered ring and different stereogenic elements.

### 2.1. Procter's synthesis of briarane 10-membered ring of solenolide F

**Figure 4.**
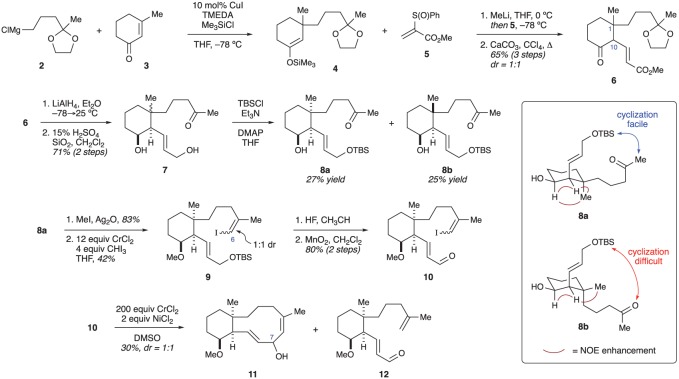
Procter's approach to the bicyclo[8.4.0]tetradecane ring system.

In 1995, Procter and co-workers reported the first synthetic work related to the briaranes (figure 4) [14]. Their efforts focused specifically on devising an efficient route to the bicyclo[8.4.0]tetradecane ring system. To accomplish this, they borrowed chemistry from Leyendecker and Comte, who found that vinyl sulfoxides could serve as an effective Michael acceptor for enolates [15]. Procter's work began with the Cu-catalysed conjugate addition of Grignard reagent **2** into enone **3**. The resulting enolate was trapped with Me_3_SiCl to afford silyl enol ether **4**. Addition of methyl lithium to compound **4** generated the corresponding lithium enolate, which engaged sulfoxide **5** as planned. After elimination, ketone **6** could be obtained in good overall yield, but as a 1 : 1 mixture of diastereomers at C1 and C10. Reduction of the ketone and ester groups, and hydrolysis of the ketal protecting group, provided diol **7**. Protection of the primary alcohol as the *tert*-butyldimethylsilyl (TBS) ether allowed the two diastereomers (**8a** and **8b**) to be separated chromatographically. The relative configuration of **8a** and **8b** was assigned using coupling-constant analysis and a series of nuclear Overhauser effect (NOE) experiments. The authors hypothesized that **8a** would more readily undergo the required cyclization due to the *trans*-diaxial conformation of **8b**, which would position the two sidechains far from each other (figure 4, box). Consequently, only diastereomer **8a** was advanced through the synthetic sequence. This proved to be an important early insight into the conformational constraints surrounding the macrocyclization (see §4 for a similar analysis).

After protection of the secondary alcohol in **8a** as an ether, a Takai olefination was used to convert the ketone into the vinyl iodide needed for ring closure. Unfortunately, iodide **9** formed as an inseparable 1 : 1 mixture of *E* and *Z* isomers. Subsequent removal of the TBS ether and oxidation to the aldehyde gave cyclization precursor **10**. After optimization, the authors found that cyclized product **11** could be obtained in low yield (1 : 1 dr at C7) by using a massive excess (200 equivalents) of CrCl_2_ in dimethylsulfoxide. The desired product was accompanied by ‘substantial amounts’ of a compound (**12**) in which the iodide had been replaced by a proton. It was hypothesized that the *E*-vinyl iodide gives rise to this material, as it is unable to cyclize. Although Procter and co-workers were able to successfully form the 10-membered ring, the poor diastereoselectivity at C1, C6 and C10, as well as the use of extremely large amounts of toxic, expensive CrCl_2_, made this route unsuitable for further optimization. No further studies by the Procter laboratory have been reported.

### 2.2. Nantz's synthesis of solenolide F fragments

**Figure 5.**
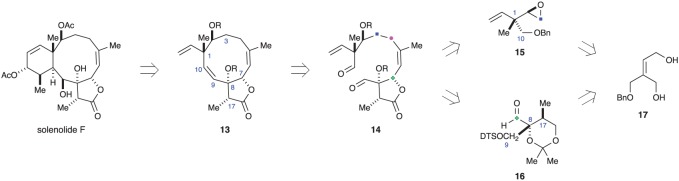
Nantz's retrosynthetic analysis of solenolide F (DTS = dimethylthexylsilyl).

In 1997, Nantz and co-workers reported synthetic work inspired by solenolide F [16]. As outlined in figure 5, they planned to initially construct the 10-membered ring and use compound **13** as an intermediate onto which the six-membered ring would be appended. They envisioned compound **13** arising from a McMurray coupling of dialdehyde **14**. Recognizing that the two fully substituted carbon atoms in **13** (C1 and C8) both have adjacent oxygenated stereogenic carbon atoms (C2 and C7), they hypothesized that the skeleton could be broken into three fragments. Two of these fragments (**15** and **16**) contain all of the required stereochemical information. The third was an unidentified, four carbon fragment that could function as a nucleophile on both ends (referred to by the authors as a ‘propyne equivalent’). Interestingly, the authors proposed that fragments **15** and **16** could both be accessed through regioselective ring opening of an epoxide with a methyl anion equivalent. Furthermore, they believed that the epoxides needed to form **15** and **16** could both be derived from a compound similar to diol **17**.

**Figure 6.**
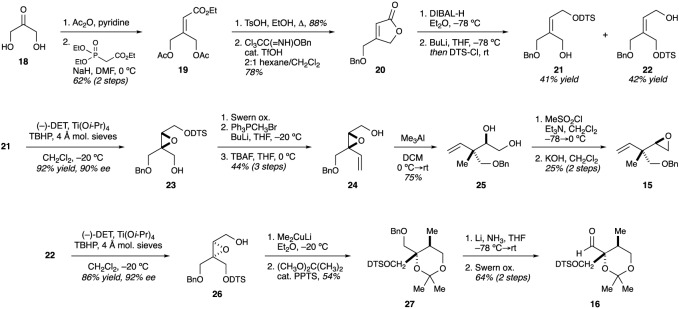
Nantz's synthetic route to briarane building blocks.

The authors began by synthesizing the diol precursor to both **15** and **16** (figure 6). Commercially available 1,3-dihydroxyacetone (**18**) was acetylated, and the resulting bis-acetate converted to triester **19** by means of a Horner–Emmons olefination. Cleavage of the acetates under acidic conditions led to spontaneous lactonization. The remaining primary alcohol was then benzylated to give **20** in good yield. Reduction of the lactone provided a diol that was subjected to mono-silylation with dimethylthexylsilyl chloride (DTS-Cl). After a survey of protecting groups, the authors found that the DTS group led to mono-silylated products **21** and **22** in approximately equal amounts. This would prove ideal, as alcohols **21** and **22** are useful for the preparation of different fragments [16].

Alcohol **21** was subjected to a Sharpless asymmetric epoxidation to give epoxide **23** in good yield and enantiomeric purity. Swern oxidation of the primary alcohol and Wittig olefination gave vinyl epoxide **24**, after removal of the DTS group. Based on results obtained in a series of model reactions, ring-opening of epoxide **24** was accomplished with trimethylaluminium. The authors were pleased to discover that desired diol **25** was the major product. They proposed that the benzyl protecting group in **24** minimized S_N_2′ addition, while the adjacent π-system served to minimize migration of the neighbouring groups. Finally, activation of the primary alcohol in **25**, followed by treatment with KOH, formed fragment **15** [16].

A Sharpless asymmetric epoxidation was also performed on alkene **22**, to give epoxide **26**. A methyl group was added to the less hindered carbon of the epoxide using Me_2_CuLi. The resulting diol was then protected as acetonide **27**. Finally, a Birch reduction removed the benzyl protecting group and a Swern oxidation produced aldehyde fragment **16** in good yield [16]. Ultimately, the Nantz group was able to construct both of the planned building blocks. Unfortunately, neither the union of fragments **15** and **16** nor the subsequent closure of the 10-membered ring have been reported.

## 3. Approaches to the central stereotetrad

In more recent years, synthetic efforts have been focused on constructing what is arguably the most challenging portion of the briaranes—the C1–C2–C10–C14 stereotetrad. Not only does this fragment include a stereogenic, all-carbon quaternary carbon; but it is flanked by three additional stereocentres. Correctly setting the relative configuration of these four contiguous stereocentres represents a major synthetic challenge, and attempting to ‘correct’ the configuration of any one of these will require additional synthetic steps to what will likely be an already lengthy synthesis. Nevertheless, as this structural motif is present in a majority of briarane family members, the rapid construction of the stereotetrad represents an important strategic opportunity. One could imagine that a well-designed fragment containing the complete stereotetrad might be able to serve as a building block for several different family members. This idea still has yet to be realized, though several groups have reported significant progress in this area.

### 3.1. Ito and Iguchi's synthesis of the briarane stereotetrad

**Figure 7.**
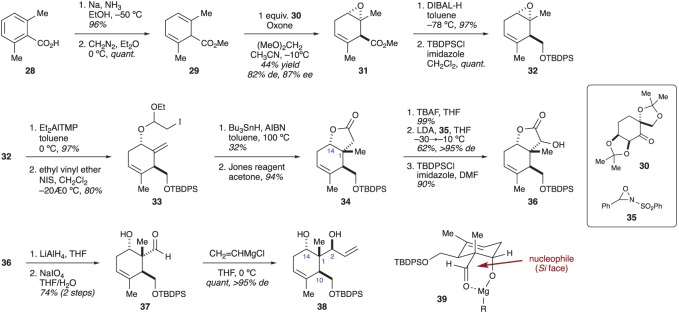
Ito and Iguchi's synthetic route to a potential briarane building block.

In 2006, the Ito/Iguchi laboratory reported the first synthetic approach to the briarane stereotetrad [17]. This effort was driven by a desire to synthesize pachuclavulide B, an anticancer briarane family member isolated by the same researchers [18]. More specifically, they hypothesized that compound **38** (figure 7) could serve as a key intermediate, upon which the 10-membered ring could be appended. In examining this potential substructure, the authors identified latent symmetry within the molecule, which they hoped to exploit. To that end, they postulated that **38** could be derived from epoxide **31**, which would come from 2,6-methylbenzoic acid (**28**).

The authors began their synthesis by performing a Birch reduction of 2,6-dimethylbenzoic acid, followed by conversion to methyl ester **29**. After some optimization, they found that the desymmetrization of diene **29** was best carried out using a stoichiometric amount of d-sorbose-derived catalyst [19] **30**. Under these conditions, epoxide **31** could be obtained in 44% yield with 81% de and 87% ee. Ester **31** was then reduced with DIBAL-H and the resulting alcohol protected to give silyl ether **32**. Recrystallization at this stage improved the enantiopurity to 99% ee. Epoxide **32** was then converted to iodo acetal **33** over two steps. A radical cyclization was then used to construct the all-carbon quaternary carbon at C1. The formation of the C1 stereocentre was, presumably, dictated by the configuration at C14. Unfortunately, the desired reaction pathway (5-*exo*-*trig*) was accompanied by significant amounts of iodine-reduction and 6-*endo*-*trig* cyclization. Nevertheless, the authors elected to carry the material forward [17].

Having formed three of the four stereocentres, the authors turned their attention to generating the aldehyde needed to form the C2 stereocentre. In order to accomplish this transformation, one carbon would need to be excised. To this end, a Jones oxidation was used to convert the radical cyclization product into lactone **34**. The direct, α-hydroxylation of lactone **34** proved to be quite difficult and the authors speculated that the bulky *tert*-butyldiphenylsilyl (TBDPS) group was the culprit. Based on this hypothesis, the silyl ether was removed. Subsequent hydroxylation of the lactone enolate with Davis' oxaziridine (**35**) proceeded smoothly and provided lactone **36** after reinstallation of the TBDPS ether. Reduction of lactone **36** with LiAlH_4_ and oxidative cleavage of the diol afforded aldehyde **37**. Finally, addition of vinylmagnesium chloride led to the isolation of diol **38** in quantitative yield as a single diastereomer. The authors confirmed the stereochemistry through NOE correlations of the corresponding acetonide (not shown). The authors proposed a chelation model (**39**) in order to rationalize the high level of stereocontrol. Coordination with the magnesium alkoxide of the C14 hydroxyl group would lock the aldehyde in a conformation wherein one face is effectively blocked by the TBDBS group. This leads to selective addition of the Grignard reagent from the *Si* face [17]. Through this route, the authors were able to access the stereotetrad core of the briarane diterpenoids, in enantioenriched form, in 18 steps from 2,6-methylbenzoic acid. Also notable is their incorporation of a double bond between C11 and C12. Many briarane family members contain a double bond at this position, or have functionality (hydroxyl groups, epoxides) that could arise from an alkene in this position. No further synthetic efforts from this group have been reported.

### 3.2. Bates’ synthesis of the briarane stereotetrad

**Figure 8.**
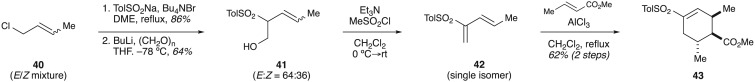
Construction of the six-membered ring using a Diels–Alder reaction.

As in the previous report by Ito and Iguchi, Bates and co-workers identified that a subunit containing an intact stereotetrad could serve as a common building block for numerous family members. Their approach hinged on setting the stereocentre at C1 through a sigmatropic rearrangement, and the use of a Diels–Alder reaction to form the six-membered ring [20]. The authors began by synthesizing a diene that would not only react with the planned crotonate dienophile, but also provide for facile installation of the allylic alcohol needed for the planned sigmatropic rearrangement. To this end, sodium *p*-toluenesulfinate was alkylated with crotyl chloride (**40**) under phase-transfer conditions (figure 8). The resulting sulfone was then converted into *β*-hydroxysulfone **41**. Elimination of the alcohol was effected using MsCl and NEt_3_. The resulting diene (**42**) was prone to spontaneous dimerization when kept in its pure form, but could be stored at –78°C as a solution in CH_2_Cl_2_. A Lewis acid-mediated Diels–Alder reaction between diene **42** and methyl crotonate was then used to access cyclohexene **43** [20]. This reaction is notable for being an uncommon example of a [4 + 2] cycloaddition involving reaction partners that are both electron-deficient [21]. An additional complicating factor is the reluctance of crotonates to participate as dienophiles. Consequently, extended reaction times (7 days) at elevated temperatures were necessary to achieve high conversions. Nevertheless, the reaction proceeded smoothly and Diels–Alder adduct **43** was obtained as a single diastereomer.

**Figure 9.**
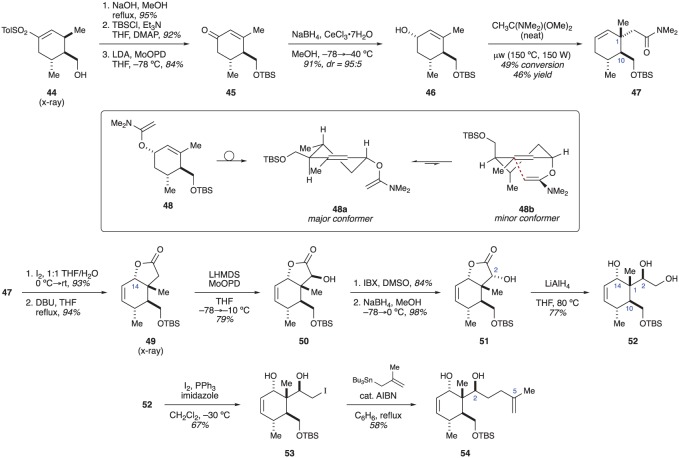
Bates’ synthesis of a potential briarane building block.

The authors found removal of the sulfone to be quite challenging. Numerous oxidative methods were evaluated with little success. Finally, it was discovered that the double bond in alcohol **44** (prepared by reduction of ester **43**) could be isomerized by heating under basic conditions (figure 9). After TBS protection of the alcohol, the sulfone was then oxidatively removed with lithium diisopropylamide and MoOPD (MoO_5_•Py•DMPU) [22] to give enone **45** along with a small amount of over-oxidized products. Luche reduction of the enone provided equatorial alcohol **46**. The allylic alcohol proved to be quite recalcitrant to the planned sigmatropic rearrangement. Eventually, the authors found an Eschenmoser–Claisen rearrangement to be effective. Thus, treating allylic alcohol **46** with dimethylacetamide dimethylacetal in xylene under microwave irradiation gave desired product **47** in moderate, but acceptable yield [20]. The difficulties experienced during the various attempts at using a sigmatropic rearrangement to generate the C1 stereocentre can be attributed to unfavourable conformational restraints in ketene aminal **48**, the reactive species in the rearrangement. Two half-chair conformers are especially germane to our discussion. Conformer **48a** is likely the most favoured as all groups can be placed in equatorial positions. However, in this conformation, the ketene aminal moiety is not appropriately positioned to engage the alkene for the rearrangement. By contrast, conformer **48b** places the ketene aminal in an appropriate position (axial) to react with the alkene, but all of the other groups will also be in an axial position, making conformer **48b** higher in energy than **48a**.

With the C1 and C10 stereocentres established with the correct relative configuration, the authors' attention turned to installing the C2 and C14 stereocentres. To this end, an iodolactonization was performed on amide **47**, establishing the C14 stereocentre. Elimination of the resulting iodide with 1,8-diazabicyclo[5.4.0]undec-7-ene provided alkene **49**, the assignment of which was confirmed by X-ray crystallography. α-Oxygenation of lactone **49** with lithium bis(trimethylsilyl)amide (LiHMDS) and MoOPD then gave alcohol **50**. Unfortunately, the C2 stereocentre in compound **50** was formed with the configuration opposite to what is required by the natural products. Consequently, a two-step sequence was needed to invert the configuration. This was accomplished through oxidation of alcohol **50** with 2-iodoxybenzoic acid and reduction of the ketone with NaBH_4_. With the C2 stereocentre in place, lactone **51** was reduced with LiAlH_4_ to provide triol **52**. The primary alcohol in **52** could be selectively converted to an iodide (**53**) by treating with I_2_ and PPh_3_. The remainder of the side-chain was installed through a Keck allylation to give briarane building block **54** in 16 steps [20]. No further progress has been reported by the Bates laboratory. Compared to compound **38** synthesized by Ito and Iguchi, the building block constructed by Bates contains a C2–C5 fragment that is well positioned for eventual ring closure; presumably through a ring-closing metathesis (RCM) reaction. However, the placement of the alkene in the six-membered ring of **54** is, arguably, not as ideal as that seen with compound **38**.

### 3.3. Harned's synthesis of the briarane stereotetrad

In 2015, Moon & Harned reported a very rapid entry into the briarane stereotetrad [23]. Their approach was predicated on the densely functionalized nature of, and the predictable reactivity patterns afforded by, 2,5-cyclohexadienones. Furthermore, in contrast to the work of Ito/Iguchi and Bates, the reaction Harned and Moon used to generate the C1 stereocentre was not directed by an existing C14 stereocentre.

**Figure 10.**
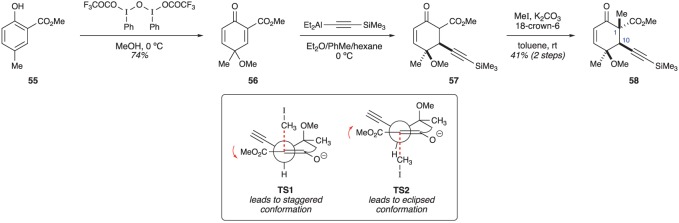
Harned's formation of the C1 and C10 stereocentres.

Oxidation of methyl salicylate **55** with the indicated μ-oxobridged diiodide provided cyclohexadienone **56** in good yield (figure 10). Subsequent conjugate addition of an aluminium acetylide into the more electron-deficient alkene generated β-ketoester **57**. In order to avoid potential rearomatization reactions, intermediate **57** was immediately alkylated with methyl iodide. The moderate overall yield of the conjugate addition/alkylation sequence was offset by the significant increase in molecular complexity the sequence provided. Compound **58** was isolated as a single diastereomer containing the correct relative configuration at C1 and C10 [23]. Molecular modelling calculations were performed to better understand the factors influencing the stereochemical outcome of the alkylation reaction [24]. To a first approximation, the stereoselectivity is consistent with Houk's torsional steering model [25]. In this case, **TS1** is responsible for forming the observed stereoisomer and is favoured as it leads to a product with a staggered conformation. In contrast, diastereomeric transition state **TS2** leads to an eclipsed conformation, and was found to be higher in energy.

**Figure 11.**
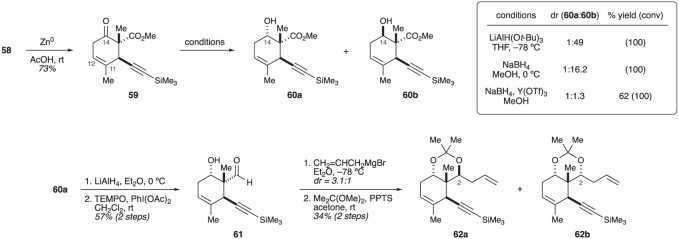
Harned's completion of the briarane stereotetrad.

Having formed the C1 and C10 stereocentres, the authors' attention turned to the stereoselective reduction of the C14 ketone (figure 11). First, enone **58** was treated with metallic zinc in order to effect the reductive cleavage of the methoxy group at C11. Many conditions were screened for their ability to convert ketone **59** into desired alcohol **60a**. Simple reducing agents, such as LiAlH(O*t*-Bu)_3_ and NaBH_4_, revealed a strong preference for forming the undesired diastereomer (**60b**). Fortunately, including stoichiometric Y(OTf)_3_ as a Lewis acidic additive with NaBH_4_ provided both diastereomeric alcohols in roughly equal amounts [23]. At the time, the strong preference for forming **60b** was seen as a nuisance. However, subsequent work by Crimmins (§4) has revealed that **60b** may eventually prove useful.

With alcohol **60a** in hand, the authors then set out to install the final stereocentre at C2. Based on the work of Ito/Iguchi (§3.1), it was anticipated that addition of a suitable organometallic reagent into β-hydroxyaldehyde **61** would lead to the selective formation of desired diastereomer **62a** (after acetonide formation). In practice, the addition of allylmagnesium bromide was poorly selective and provided a diastereoselectivity of about 3 : 1 under optimal conditions. The authors identified the strong axial preference of the alkyne as one contributing factor for the poor selectivity. Such a conformational preference might disrupt the preferential chelation that was the basis of the model proposed by Ito/Iguchi [23]. At the same time, the high reactivity displayed by allyl Grignard reagents [26] may also contribute to the diminished stereoselectivity. Although further work is required to address the different stereochemical problems associated with this route, it illustrates that the densely functionalized nature of cyclohexadienones can be leveraged into a very rapid construction of the requisite stereotetrad.

## 4. Construction of the *trans*-fused ring system

In 2017, Crimmins *et al.* reported the most significant progress to date in the quest to develop a synthetic route to the briarane diterpenoids [27]. In this work, they were able to successfully form the bicyclo[8.4.0]tetradecane ring system; however, unlike Procter's work [14], Crimmins was able to access the 6,10 ring system with a complete stereotetrad. Although, the Crimmins work would ultimately fall short of realizing a completed total synthesis, it serves as an important foundation for future work by uncovering important reactivity patterns that will prove useful in future synthetic efforts. By disconnecting at the C1–C10 bond, Crimmins and co-workers took a very different approach to forming the all-carbon quaternary stereocentre.

**Figure 12.**
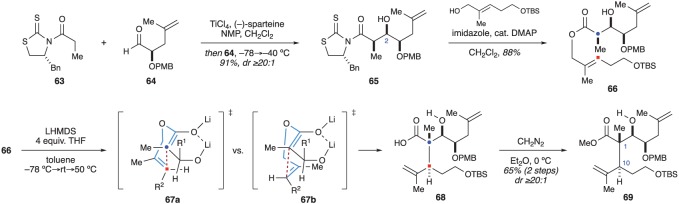
Crimmins’ use of a dianionic Ireland–Claisen rearrangement to set the C1 and C10 stereocentres.

The authors began with thiazolidenthione **63**, which was employed in an asymmetric aldol reaction with aldehyde **64** (figure 12). Aldol product **65** was then converted into ester **66** upon treatment with the indicated alcohol under basic conditions. Based on methodology developed by the Kurth [28] and Crimmins [29] groups, double deprotonation of ester **66** with LiHMDS initiated a dianionic Claisen rearrangement to deliver carboxylic acid **68**, which was immediately converted into ester **69** using diazomethane. The rearrangement proceeded with excellent stereocontrol and ester **69** was obtained as a single stereoisomer [27]. The high degree of stereocontrol was rationalized by considering transition states **67a** and **67b**. Of these two, chair-like transition state **67a** was expected to be favoured, as boat-like transition state **67b** places the alkenyl methyl group and the R^2^ group in unfavourable steric environments. Similarly, the R^1^ group of the chelated enolate forces the rearrangement to occur on only one face of the enolate [28,29].

**Figure 13.**
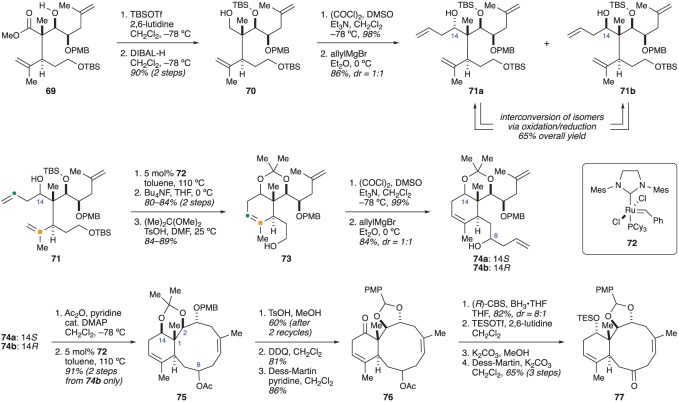
Crimmins’ use of ring-closing metathesis to form the 6,10 ring system of the briaranes.

With the C1 stereocentre set, Crimmins and co-workers turned their attention to constructing the six-membered ring (figure 13). The secondary alcohol in compound **69** was protected as a TBS ether and the ester was reduced to primary alcohol **70**. Swern oxidation and addition of allylmagnesium bromide provided alcohol **71** as a 1 : 1 mixture [26] of diastereomers. Fortunately, alcohols **71a** (corresponding to the natural configuration at C14) and **71b** could be separated by column chromatography and could be recycled in either direction though an oxidation/reduction sequence. The two alcohols were separately carried though a five-step sequence in order to access cyclohexene **74**. First, a RCM was performed by exposing alcohol **71** to the second-generation Grubbs catalyst. Cleavage of the silyl ethers and selective protection of the resulting 1,3-diol gave acetonide **73**. Swern oxidation of the primary alcohol and addition of allylmagnesium bromide then provided alcohols **74a** (from **71a**) and **74b** (from **71b**) as a mixture of stereoisomers at C8 [27].

**Figure 14.**
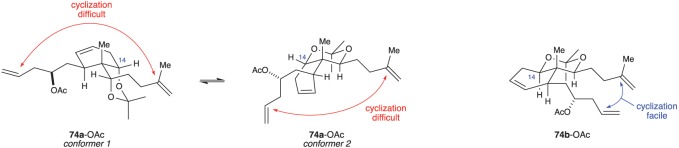
Crimmins’ use of ring-closing metathesis to form the 6,10 ring system of the briaranes.

With acetonides **74a** and **74b** in hand, the authors were poised to close the 10-membered ring. For this, they planned to carry out another RCM reaction. Considering the limited number of examples of RCM being used to form 10-membered carbocyclic rings [30–32], this would be a difficult task. Curiously, the C8 acetate derived from acetonide **74a** failed to undergo ring closure, even when other protecting groups were used instead of the acetonide. However, the C8 acetate derived from acetonide **74b** smoothly reacted to give compound **75** in 91% yield. Much like the analysis used by Procter (§2.1), Crimmins and co-workers rationalized the reactivity, or lack thereof, of acetonides **74a** and **74b** by considering their conformational preferences (figure 14). They thought that substrate **74a**-OAc (*14S*) might adopt one of two conformers, both of which place the ends of the two alkene chains far away from each other. In contrast, substrate **74b**-OAc (*14R*) is able to adopt a conformation in which the two alkene chains are proximal to one another, thereby facilitating ring closure [27].

Unfortunately, the configuration at C14 in compound **75** does not correspond to the natural stereocentre. Fortunately, this could be remedied by first replacing the acetonide between C2 and C14 with an acetal between C2 and C3. Oxidation of the C14 hydroxyl group delivered ketone **76**. Stereoselective reduction of the ketone restored the C14 stereocentre, now with the correct configuration. Reprotection of the C14 hydroxyl group was followed by cleavage of the C8 acetate and oxidation to ketone **77**. Seemingly, the C8 ketone present in compound **77** is well positioned for installation of the lactone present in many briarane family members. However, none of the attempts at generating the butenolide from the corresponding α-hydroxyketone were fruitful (details not provided) [27]. Although this was a setback to Crimmins and co-workers, this observation is of critical importance for future synthetic endeavours as it suggests that the C7–C8 γ-lactone may need to be generated before closing the 10-membered ring.

## 5. Conclusion

In conclusion, the briarane diterpenoids are an intriguing class of bioactive synthetic targets. Several exploratory routes have been reported with varying degrees of success. Although this work has yet to produce a completed total synthesis, several key observations have been made that will undoubtedly inspire future efforts in this area. For example, Crimmins' successful use of an RCM to close the ten-membered ring, and the associated stereochemical constraints, is a major development that will likely be an important disconnection moving forward. At the same time, the problems experienced by Crimmins and co-workers when trying to install the C7–C8 lactone at a late stage is a major impediment that will need to be addressed in the future.

**Figure 15.**
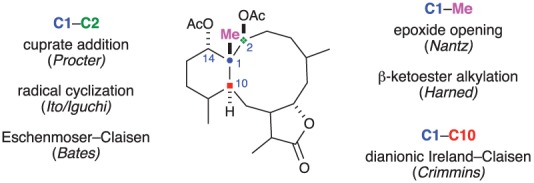
Summary of strategies used to construct the C1 stereocentre.

As mentioned earlier, the C1–C2–C10–C14 stereotetrad arguably represents the biggest synthetic challenge associated with the briarane family. Accessing this motif quickly and with minimal corrections to the relative stereochemistry will be crucial in order to realize an efficient and tractable synthesis. As summarized in figure 15, three different C–C bond disconnections have been used to construct the stereogenic quaternary carbon at C1. Other disconnections likely will be reported in the future, and it will be interesting to see what advantages they offer. It will be interesting to see how future work makes constructive use of the conformational preferences of ring-closure explored by Procter and Crimmins.

## Data accessibility

This article has no additional data.
